# Awareness and knowledge of developmental coordination disorder: A survey of caregivers, teachers, allied health professionals and medical professionals in Australia

**DOI:** 10.1111/cch.12824

**Published:** 2020-11-16

**Authors:** Jacqui Hunt, Jill G Zwicker, Erin Godecke, Annette Raynor

**Affiliations:** ^1^ School of Medical and Health Sciences Edith Cowan University Joondalup Western Australia Australia; ^2^ Department of Occupational Science & Occupational Therapy University of British Columbia Vancouver British Columbia Canada; ^3^ Department of Pediatrics University of British Columbia Vancouver British Columbia Canada; ^4^ Brain, Behaviour, & Development BC Children's Hospital Research Institute Vancouver British Columbia Canada; ^5^ Neuromotor Program Sunny Hill Health Centre at BC Children's Hospital Vancouver British Columbia Canada

**Keywords:** awareness, DCD, developmental coordination disorder, diagnosis, knowledge

## Abstract

**Background:**

To allow for accurate and timely diagnosis of developmental coordination disorder (DCD) key stakeholders must be familiar with and be able to identify features of this disorder. No studies to date have investigated the awareness of DCD among key stakeholders in Australia.

**Methods:**

An online survey was complete by 494 Australian participants: primary caregivers (*n* = 153), teachers (*n* = 149), allied health professionals (*n* = 165) and medical professionals (*n* = 27).

**Results:**

DCD and related terms were among the least known childhood disorders. Approximately half of the sample were familiar with the term DCD but every stakeholder group were more familiar with the term dyspraxia. Allied health professionals demonstrated greater knowledge of the features of DCD, particularly motor features. Every stakeholder group showed poor recognition of the social and psychological effects of DCD. A relatively low percentage of allied health (53%) and medical (33%) professionals reported they had identified or diagnosed DCD and less than 20% of these felt that the DSM‐5 contained adequate information to make a DCD diagnosis. Most teachers (82%) believed they should play a role in identifying early warning signs of this disorder, and 80% believed there are children in the school system who were labelled as lazy or defiant when they have motor skills impairments. Primary caregivers were supportive of a diagnosis of DCD being provided; however, only 16% were confident that a physician would provide an accurate and timely diagnosis.

**Conclusion:**

Key stakeholders play a unique and important role in the identification of children with DCD. Though most participants acknowledge the role that they play, all stakeholder groups demonstrated poor familiarity with the term DCD and low levels of knowledge about the features of this disorder. Improved familiarity and knowledge of the disorder is needed for access to appropriate services and improved long‐term outcomes for this condition.

**Key Messages**

- Despite affecting one in 20 children, DCD is one of the least familiar childhood conditions among Australian parents, teachers, allied health and medical professionals.
- Most stakeholders were unaware of the impact of DCD on mental health (e.g., anxiety and depression) and quality of life.
- Ongoing knowledge translation is necessary to raise awareness and increase supports and services for children with DCD.

## 1 INTRODUCTION

Affecting one in 20 children, developmental coordination disorder (DCD) is a common but underrecognized neurodevelopmental disorder characterized by impaired ability to acquire and execute coordinated motor skills quality (American Psychiatric Association, 2013). DCD significantly interferes with activities of daily living, school performance, leisure pursuits and play. Secondary psychosocial issues are common, including increased externalizing (e.g., frustration and aggression) and internalizing behaviours (e.g., anxiety and depression) compared with children without motor deficits (Crane, Sumner, & Hill, 2017; King‐Dowling, Missiuna, Rodriguez, Greenway, & Cairney, 2015). The motor and psychosocial sequelae of DCD have a significant impact on children's quality of life (Gagnon‐Roy, Jasmin, & Camden, 2016; Zwicker, Harris, & Klassen, 2013; Zwicker, Suto, Harris, Vlasakova, & Missiuna, 2018) and tend to persist into adulthood (Cousins & Smyth, 2003; Kirby, Sugden, & Purcell, 2014; Kirby, Williams, Thomas, & Hill, 2013; Timler, McIntyre, Cantell, Crawford, & Hands, 2016).

DCD is well defined in the Diagnostic and Statistical Manual (fifth ed.) (DSM‐5) which specifies the following four diagnostic criteria: (a) motor skills acquisition and execution are significantly below age‐matched peers, despite opportunities for learning and using these skills; (b) motor difficulties significantly and persistently interfere with age‐appropriate activities of daily living, school and play; (c) symptoms begin during early childhood development; and (d) difficulties cannot be attributed to other conditions, such as intellectual disability, visual impairment or other neurological disorders that affect movement (American Psychiatric Association, 2013). The prevalence of DCD is approximately 5–6% of children (Blank et al., 2019) and multiple terms such as dyspraxia, clumsy child syndrome, motor learning difficulty, minimal brain dysfunction, sensory integration disorder and disorder of attention and motor perception (DAMP) have been used to describe this disorder (Gibbs, Appleton, & Appleton, 2007). Inconsistent terminology in clinical practice and in research is a barrier to accurate identification of this condition and is likely to have contributed to poor estimates in prevalence and poor comparability and knowledge translation in this field (Magalhães, Missiuna, & Wong, 2006; Polatajko, Fox, & Missiuna, 1995).

Issues of nomenclature were addressed at a consensus meeting of DCD experts in London, Ontario, Canada, in 1994. The international panel recommended the preferential use of DCD to describe children with significant difficulties in motor coordination (Polatajko et al., 1995). DCD does not appear to be well understood by relevant stakeholders, including primary caregivers, teachers and allied health/medical professionals, despite this consensus, sound knowledge of the impact of this disorder, high incidence and clear diagnostic criteria. (Harris, Mickelson, & Zwicker, 2015; Wilson, Neil, Kamps, & Babcock, 2012).

In a sample of key stakeholders, Wilson et al. (2012) found that only 20% of parents, teachers and medical professionals had knowledge of DCD, highlighting the need for improved awareness of the condition. International recommendations for the definition, diagnosis and management of DCD were published in 2012 (Blank et al., 2012) and updated in 2019 (Blank et al., 2019). It is unclear if either version of these guidelines has improved the recognition of DCD among relevant stakeholders.

An estimated 25% of the children with the condition are identified prior to starting school (Gibbs et al., 2007), due to delayed developmental milestones, (e.g., crawling, walking and speech) or significant difficulties with self‐care activities, poor ball skills or immature drawing. Delays in these early developmental milestones are not always evident and consequently; identification is more common in the first years of primary school, when parents and teachers recognize that the child is significantly behind their peers and not making necessary improvements in complex skills, such as handwriting and sports (Gibbs et al., 2007; Missiuna, Rivard, & Campbell, 2017). Due to large variations in typical motor development, it is recommended that a DCD diagnosis only be given to children under 5 years of age in the case of severe difficulties (Blank et al., 2019).

Despite parents, teachers and medical practitioners in the United States, United Kingdom and Canada having a poor of knowledge of DCD (Wilson et al., 2012), this has yet to be established in the Australian context. This study aims to examine the current knowledge and perceptions of DCD among key stakeholders in Australia. Specifically, the study compared levels of familiarity with, and knowledge of, DCD across different stakeholder groups.

## 2 ETHICS

thical approval (No. 2019‐00106‐HUNT) was obtained from the Human Research Ethics Committee of Edith Cowan University. All participants provided informed consent prior to commencing the study.

## 3 METHODS

A quantitative cross‐sectional survey (see Appendix S1) was adapted with permission (Wilson et al., 2012) and was distributed online for an 8‐week period from August to October 2019, using Qualtrics.

### 3.1 Participants

A recruitment flyer containing a link to the survey was distributed throughout Australia via social media and e‐mail to relevant professional associations, schools, paediatric and general medical practices and therapy providers to recruit primary caregivers, teachers, allied health and medical professionals. Recipients were asked to share the survey link, enabling snowball sampling.

Participants were required to be residents of Australia, able to complete the survey in English and care for or work with children (<19 years old). Teachers with experience of working within this age range were eligible to participate, as were health professionals with experience working in a caseload of at least 15% children. All professionals were required to hold a current registration with the relevant registration board either in their state/territory or nationally.

### 3.2 Data collection

The survey was piloted in Western Australia with a sample of 223 participants (Falck, 2018), with changes subsequently made to the demographic sections of the survey to allow respondents from across Australia to participate. A response option of ‘unsure’ was also added to all knowledge questions.

The survey contained four sections. Section A collected demographic information and determined stakeholder eligibility and category. Participants then answered only those questions related to their specific stakeholder group. If a participant met the criteria for more than one stakeholder category (i.e., parent and professional), they were placed into their professional category for data analysis, assuming that the greatest knowledge of childhood conditions would come from their professional role. If a stakeholder was a professional and a parent but did not meet inclusion criteria for the professional group, they were placed in the primary caregiver category.

In section B, participants were required to rate their level of familiarity with 18 childhood conditions on a 5‐point Likert scale: ‘I have not heard of this condition at all’, ‘very unfamiliar’, ‘somewhat unfamiliar’, ‘somewhat familiar’ or ‘very familiar’. The list of conditions was based on those included in the study of Wilson et al. (2012) and incorporated both older terms (such as Asperger's syndrome and clumsy child syndrome) and current nomenclature. If participants indicated that they were ‘somewhat’ or ‘very familiar’ with DCD, dyspraxia, motor learning disability or clumsy child syndrome, they were directed to section C of the survey. This section examined participants' knowledge of DCD on a 4‐point Likert scale; participants were asked if they thought particular motor, social and cognitive features were either a ‘common feature of DCD’, ‘may be a feature of DCD’, ‘not part of the condition of DCD’ or ‘unsure’. Section D explored stakeholder perceptions and opinions about children with DCD and current levels of education about this disorder. Using a 3‐point Likert scale of ‘agree’, ‘disagree’ and ‘unsure’, participants were asked a range of questions about current levels of research and services for children with DCD and collaboration among stakeholder groups. To enhance the insight gained from the quantitative information, the questionnaire concluded with an open‐ended question: ‘What are the major factors that influenced your answers to the above questions?’

### 3.3 Data analysis

All data were analysed using descriptive and non‐parametric statistics to compare levels of familiarity with, and knowledge of, DCD across different stakeholder groups. Continuous variables (demographics) were reported as median and range (min, max) and analysed with the Kruskal–Wallis *H* test. Chi‐square tests with pairwise post hoc comparisons were used to analyse the association between level of familiarity (categorical data) across stakeholder groups. The level of significance was set at *α* < 0.05 with Bonferroni correction for multiple comparisons. The open‐ended question was analysed using content analysis to supplement the quantitative findings (Lindgren, Lundman, & Graneheim, 2020).

## 4 RESULTS

### 4.1 Participant demographics

**FIGURE 1:**
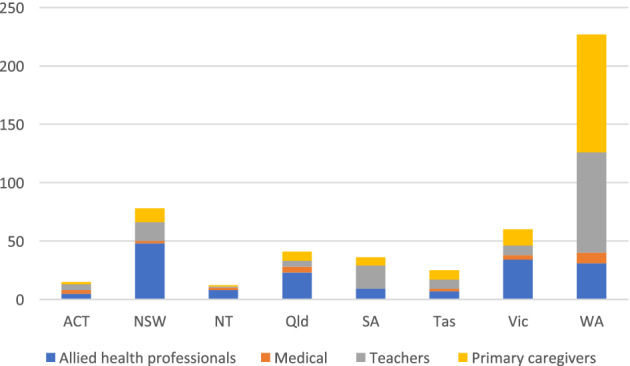
Residency of stakeholder groups. ACT, Australian Capital Territory; NSW, New South Wales; NT, Northern Territory; Qld, Queensland; SA, South Australia; TAS, Tasmania; VIC, Victoria; WA, Western Australia

A total of 581 respondents agreed to participate in the survey, with 87 excluded as they did not meet inclusion criteria (i.e., caregivers with children over 20 years, nonregistered practitioners, teachers or professionals who had never worked with at least 15% of children in their caseload). A total of 494 participants completed the survey with the distribution by stakeholder group shown in Table 1. Allied health professionals were the largest group, with over 70% being occupational therapists. The residency of all respondents is presented in Figure 1 with all states represented. As expected, the largest cohort was from Western Australia (the researcher's state of residence).

**TABLE 1:** Stakeholder groups and years of professional experience

	*n* (% of total)	Female *n* (%)	Years of professional experience		
			Median	Q1	Q3
Allied health professionals	165 (33)	160 (97)	12	5	19.5
Occupational therapist	121 (24)				
Physiotherapist	15 (3)				
Psychologist	7 (1)				
Speech pathologist	19 (4)				
Other (radiographer, optometrist, social worker)	3 (1)				
Medical professionals	27 (6)	13 (48)	20	12	25
Registered nurse	1 (0)				
Paediatrician	18 (4)				
General practitioner	8 (2)				
Teachers	149 (30)	134 (90)	13	6	22
Primary caregivers	153 (31)	150 (98)			
Total	494 (100)	457 (92)			

The median years of experience across all professional groups was 13 (*IQR* = 6–21), with a significant difference between professional groups (*H* = 10.9, *P* = 0.004). Post hoc pairwise comparisons showed medical professionals had significantly more years of experience than allied health professionals (*P* = 0.003) and teachers (*P* = 0.031) (Table 1).

### 4.2 Stakeholder familiarity with childhood disorders

DCD and associated terms for this disorder (dyspraxia, motor learning disability and clumsy child syndrome) were among the least familiar terms across all stakeholder groups. Familiarity across stakeholder groups from most to least familiar is shown in Table 2. Teachers and parents were more familiar with older terminology for motor coordination difficulties. All stakeholder groups were more familiar with the term dyspraxia than DCD. Conversely, stakeholders were more familiar with current terms used for all other childhood conditions. Specifically, all stakeholder groups were more familiar with autism spectrum disorder and intellectual disability compared with Asperger's syndrome and mental retardation, respectively. The level of familiarity with DCD differed significantly between the stakeholder groups and for each of the associated terms for this disorder: clumsy child (*χ* ^2^(3, *N* = 494) = 18.2, *P* < 0.001), dyspraxia (*χ* ^2^(3, *N* = 494) = 48.9, *P* < 0.001), DCD (*χ* ^2^(3, *N* = 494) = 93.4, *P* < 0.001) and motor learning disability (*χ* ^2^(3, *N* = 494) = 14.5, *P* = 0.002). For each of these terms, post hoc analysis indicated the allied health group were more familiar with the term DCD compared with other stakeholder groups (*P* < 0.006).

**TABLE 2:** Percentage of participants who stated they were ‘very familiar’ or ‘somewhat familiar’ with childhood conditions

Childhood conditions	Total *n* = 494	AHP *n* = 165	MP *n* = 27	T *n* = 149	PC *n* = 153
Autism	97	99	96	98	94
Autism spectrum disorder	95	99	100	98	87
Attention deficit hyperactivity disorder	94	96	100	94	91
Learning disability	94	97	100	96	89
Intellectual disability	92	98	100	93	85
Dyslexia	92	90	89	94	91
Asperger's syndrome	90	96	96	94	80
Obsessive compulsive disorder	90	89	100	93	86
Global developmental delay	81	98	96	85	58
Spina bifida	79	79	96	77	77
**Dyspraxia**	**76**	**90**	**82**	**79**	**57**
Chromosomal disorders	74	88	96	63	63
Mental retardation	70	74	93	69	62
Oppositional Defiance disorder	68	81	82	75	46
**Motor learning disability**	**53**	**65**	**48**	**48**	**46**
**Developmental coordination disorder**	**51**	**81**	**52**	**35**	**34**
Conduct disorder	42	64	78	30	24
**Clumsy child syndrome**	**22**	**32**	**30**	**15**	**16**

*Note*. DCD and associated terms for this disorder are in bold emphasis.

Abbreviations: AHP, allied health professionals; MP, medical professionals; PC, primary caregivers; T, teachers.

Content analysis suggested that stakeholders who were most familiar with the term DCD had personal exposure to the condition through their family or friend network, with many parents discussing their own child's DCD diagnosis. A number of professional staff stated that their answers were influenced by their ‘own experience with my daughter’ (Allied Health Professional 140) or their ‘own personal journey with my child (Teacher 133)’ or the fact that ‘my nephew has the disorder’ (Medical Professional 02).

Familiarity with DCD was also reported by stakeholders with previous work experience with children with the disorder: ‘working with a child early on in my career who had DCD’ (Allied Health Professional 45) and ‘children have become my best PD [professional development] and have given me the knowledge to refer children as needed to appropriate agencies’ (Teacher 30).

Teachers, allied health professionals and physicians who were unfamiliar with the condition reported a lack of undergraduate education or limited professional development opportunities related to DCD. This is supported by comments such as ‘DCD is a relatively unknown condition in school and there isn't any professional learning done that I know of’ (Teacher 35), ‘other OTs [lack confidence] due to a lack of training/learning about dyspraxia/DCD’ (Allied Health Professional 32), ‘limited “formal” training/PD on DCD’ (Allied Health Professional 53), ‘we only cover this in one lecture of DCD at uni’ (Allied Health Professional 106) and ‘lack of education about the condition’ (Medical Professional 05).

### 4.3 Stakeholder knowledge of DCD

From a list of 15 features, participants were asked to identify whether these features were (a) a common feature of DCD, (b) may be a feature, (c) were not a part of the condition or (d) unsure. When stakeholders identified features as either (a) or (b), they were considered to have knowledge of the feature. The three features most known to all stakeholder groups were motor learning difficulties, difficulty printing/writing and gross motor and/or fine motor skills delay. Of these three features, a considerably higher percentage of allied health professionals associated these features with DCD. An across stakeholder comparison for each feature revealed a significant difference (*P* < 0.05) in the features indicated in Table 3. Post hoc analysis indicated a greater percentage of allied health professionals recognized a number of features relative to the other stakeholder groups, being motor learning difficulties (*P* = 0.005), printing/writing (*P* = 0.003), self‐esteem (*P* = 0.0004) and average cognitive ability (*P* = 0.0002). Medical professionals showed significantly less knowledge of gross/fine motor skills delay (*P* = 0.0004). The percentage of each stakeholder group who recognized the social and psychological effects of DCD were low, with the range of stakeholder knowledge at 13–16% for difficulty making friends, 11–12% for poor social skills and 7–13% for depression. Across all groups, 19–26% of participants indicated (incorrectly) that sensory processing challenges are a common nonmotor feature of DCD.

**TABLE 3:** Percentage of stakeholders in each group who correctly identified features that are a ‘common feature of the condition of DCD’ or ‘may be a feature of the condition of DCD’

	AHP *n* = 155	MP *n* = 23	T *n* = 120	PC *n* = 106
Common motor features of DCD				
Motor learning difficulties	85*	52	52	42
Difficulty printing and/or writing	75*	56	52	43
Gross motor and/or fine motor skills delay	81*	52	52	45
Common nonmotor features of DCD				
Low self‐esteem	50*	33	26	24
Poor physical fitness	38	22	23	20
Sensory processing challenges	22	19	26	20
Anxiety	23	22	17	18
Difficulty making friends	13	15	13	16
Poor social skills	11	11	12	11
Depression	7	7	13	11
May be a feature of the condition of DCD				
Poor academic performance	55	19	22	25
Average (or above average) cognitive ability	36*	11	9	10
Below average cognitive ability	41	4	6	5
Higher than average risk for suicide	36	4	6	2
Obesity	44	4	5	6

Abbreviations: AHP, allied health professionals; DCD, developmental coordination disorder; MP, medical professionals; PC, primary caregivers; T, teachers.

* *P* < 0.05.

Survey responses confirmed low levels of knowledge of DCD, with many stakeholders indicating that they had ‘no knowledge’ or ‘limited knowledge’ about the disorder. Other respondents (predominantly health professionals) shared their own (or others) uncertainty or misconceptions in statements such as ‘many teachers believe skills will come with time’ (Teacher 134) or ‘I'm not sure if it is the same as dyspraxia’ (Allied Health Professional 105), ‘sensory processing disorders can look very similar’ (Allied Health Professional 149) and the ‘incidence of DCD is low’ (Allied Health Professional 97). Medical Professional 16 appeared to question the significance of DCD, suggesting that labels such as DCD are too frequently ‘made up for normal spectrum of capabilities and behaviours’.

### 4.4 Identification of DCD

A relatively low percentage of allied health professionals (53%) reported they had identified DCD and one third of medical professionals reported they diagnosed this disorder. More than 80% of allied health and medical professionals felt that the DSM‐5 contained inadequate information to make a DCD diagnosis. Many allied health and medical professionals stated that they needed more information to either identify or diagnose this condition, respectively, adding that identification is difficult because DCD ‘can look like other conditions’ (Allied Health Professional 31) or because ‘assessment requires a multidisciplinary team that comprises of paediatric physio, OT and sometimes a paediatric neurologist, to be sure’ (Medical Professional 17).

Issues of identification were also evident in text responses where many allied health professionals cited medical professionals as ‘blocks’ to diagnosis, including statements that ‘paediatricians are either not confident to diagnose children or have never heard of DCD’ (Allied Health Professional 32) and ‘the need to find a paediatrician who is experienced and can consolidate info to give a diagnosis is the major hurdle’ (Allied Health Professional 87).

One paediatrician stated ‘I'm not sure that I agree that DCD is a medical diagnosis. I think we over medicalise children … there will always be clumsy children (and adults)’ (Medical Professional 12).

Many teachers (89%) felt that an accurate diagnosis of DCD was critical for teachers to know how to assist children with the condition and 65% believed that a lack of knowledge of DCD prevented adequate support for these children. Most (82%) believed that teachers should play a role in identifying early warning signs. The majority of teachers (80%) agreed that there were children in the school system who were labelled as lazy or defiant when they had gross and/or fine motor skills impairments. Open‐text comments included ‘other teachers have considered them (undiagnosed children) to be lazy, defiant, eccentric, etc.’ (Teacher 50), ‘I have seen a number of students considered lazy or unmotivated or defiant but appear to really struggle with writing as a physical exercise’ (Teacher 135) and

working with young people who have disengaged, I have found there is an underlying cause or reason that has been misplaced and one of these is that students are lazy when in fact they could have a condition such as DCD. (Teacher 144)

Primary caregivers were supportive of a diagnosis of DCD being provided; however, only 16% were confident that a physician would provide an accurate and timely diagnosis. One parent commented that they were ‘quite confident the education and medical system are not going to be experienced or confident managing it (DCD)’ (Parent 1). Most primary caregivers (93%) felt there should be more education about the signs of DCD, and only 3% felt that there were adequate resources for children with DCD.

## 5 DISCUSSION

This study is the first known Australian study to investigate knowledge and familiarity of DCD among primary caregivers, teachers, allied health and medical professionals. We found that more than one third of Australian primary caregivers and teachers were familiar with DCD, which is an improvement compared with the 6% of parents and 23% of teachers (in the United Kingdom, Canada and the United States) who were familiar with DCD (Wilson et al., 2012). Despite this increase, DCD and related terms remain among the least familiar diagnostic terms across all stakeholder groups, and the vast majority of stakeholders were unfamiliar with DCD. Similar to findings by Wilson et al. (2012), key stakeholders in our study were more familiar with outdated terminology, such as dyspraxia. Clinicians may well use the term dyspraxia in the knowledge that there is greater familiarity with this label across all stakeholders, but the terms dyspraxia and motor learning disability should not be used as they fail to account for the many complex features of this disorder. It is crucial that the term DCD is used and that adoption of consistent nomenclature among key stakeholders provides the first step to timely and accurate identification of DCD.

Difficulties in motor coordination are the defining features of DCD and form the basis of all DSM‐5 criteria (American Psychiatric Association, 2013). Despite this, only half of the primary caregivers, teachers and medical professionals in this study identified the common core, fine and gross motor features of DCD. Knowledge of the features of DCD is highest among Australian allied health professionals; however, this holds little value when they are likely the last stakeholder group to encounter a child with motor coordination difficulties (Gibbs et al., 2007).

Notably, every stakeholder group in our Australian sample showed poor knowledge of the social and emotional consequences of DCD, which is consistent with the finding among physicians and teachers in the study of Wilson et al. (2012). The nonmotor features of DCD, including clinically significant levels of anxiety and depression (Missiuna et al., 2014), must be considered in the treatment of children with DCD as they have considerable impact upon quality of life (Gagnon‐Roy et al., 2016; Zwicker et al., 2013; Zwicker et al., 2018). Without any intervention, the consequences of DCD are lifelong (Kirby et al., 2014); thus, it is crucial that stakeholders are aware of motor and nonmotor features of DCD.

Increased familiarity and knowledge of DCD will assist stakeholders to identify ‘who’ might require assessment, but it does not assist stakeholders in understanding the ‘how’ of DCD diagnosis. Despite clear diagnostic guidelines (American Psychiatric Association, 2013) and practice recommendations (Blank et al., 2012; Blank et al., 2019), this study shows that health professionals remain unclear about diagnostic processes. Most allied health and medical professionals surveyed identified the need for further information about DCD, and most did not feel that the DSM‐5 contained adequate information for an accurate diagnosis. Despite an identified need for further information, over half of the allied health professionals have identified probable DCD and one third of medical practitioners have diagnosed the disorder. Although there was only a small sample of medical professionals in this study, these results reflect the findings of Wilson et al. (2012) where only 23% of paediatricians and 9% of general physicians had diagnosed DCD.

Although our findings are consistent with Wilson et al.'s, 2012 study of stakeholders in Canada, the United Kingdom and the United States, these results were published 8 years ago. New knowledge has since been created and disseminated, but it appears that greater knowledge translation is needed in Australia. The Knowledge‐to‐Action Cycle proposed by Graham et al. (2006) provides a two‐stage process to facilitate translation of knowledge into practice: (1) knowledge creation and (2) the action cycle. Table 4 outlines resources (created knowledge) and dissemination strategies as a first step in the action cycle for increasing awareness of DCD among all stakeholder groups in Australia.

**TABLE 4:** Knowledge translation resources and strategies for each stakeholder group

Stakeholder group	Resources available online	Dissemination strategy
Medical professionals	DCD toolkit for paediatricians: http://www.childdevelopment.ca/DCDAdvocacyToolkit/DCDAdvocacyToolkitIntro.aspx Workshop for physicians: https://machealth.ca/programs/developmental_coordination_disorder/	Distribution of research findings and links to resources via the same channels used to distribute this survey (e.g., via social media and e‐mail to specific medical practices)
Allied health professionals	Recognizing and referring children with developmental coordination disorder: The role of (specific information available) for the physical therapist, occupational therapist, speech and language pathologist and psychologist: https://www.canchild.ca/en/diagnoses/developmental‐coordination‐disorder/dcd‐educational‐materials‐for‐home‐school‐physicians‐and‐other‐health‐professionals Boniface, Glegg, Montgomery, and Zwicker (2017) DCD Advocacy Toolkit (designed for occupational therapists but relevant for physical therapists too): http://www.childdevelopment.ca/DCDAdvocacyToolkit/DCDAdvocacyToolkitIntro.aspx Workshop for physical therapists (relevant for occupational therapists too): https://machealth.ca/programs/developmental_coordination_disorder/	Presentation at professional conferences Formation of national communities of practice Collaboration with existing groups (e.g., Telethon Kids Institute) Distribution of research findings and links to resources via the same channels used to distribute this survey (e.g., via e‐mail and posting in social media groups specific to each discipline)
Medical and allied health professionals	Missiuna, Gaines, and Soucie (2006) https://canchild.ca/system/tenon/assets/attachments/000/000/312/original/WhyEveryOfficeNeedsaTennisBall.pdf Harris et al. (2015) https://www.ncbi.nlm.nih.gov/pmc/articles/PMC4467929/ Blank et al. (2019) https://onlinelibrary.wiley.com/doi/full/10.1111/dmcn.14132 Ip, Mickelson, and Zwicker (In Press) (Paediatrics and Child Health, in press)	
Teachers	Understanding developmental coordination disorder: https://www.education.vic.gov.au/school/teachers/learningneeds/Pages/developmental‐coordination‐disorder.aspx Children with DCD: At home, at school and in the community (booklet): https://www.canchild.ca/en/resources/112‐children‐with‐dcd‐at‐home‐at‐school‐and‐in‐the‐community‐booklet CanChild M.A.T.C.H. flyers which are free to download from https://canchild.ca which include grade‐specific recommendations on how to MATCH activities to support children with DCD To Write or to Type—That is the Question! https://www.canchild.ca/en/resources/128‐to‐write‐or‐to‐type‐that‐is‐the‐question	Presentation at education conferences Distribution of research findings and links to resources via the same channels used to distribute this survey (e.g., via education‐specific social media groups) DCD‐specific professional development for post‐graduate teachers (online and in person)
Caregivers/parents	‘Does your child have DCD?’ https://www.canchild.ca/system/tenon/assets/attachments/000/000/179/original/DoesYourChild‐DCD.pdf Parent workshop about DCD: https://www.canchild.ca/en/diagnoses/developmental‐coordination‐disorder/workshops ‘Raising Children’ Australian parenting website https://raisingchildren.net.au/guides/a‐z‐health‐reference/development‐coordination‐disorder‐dcd#:~:text=About%20development%20coordination%20disorder%20(DCD)&text=Children%20with%20DCD%20are%20just,and%20is%20a%20lifelong%20condition Western Australian developmental OT or DOT (WA) information sheet: https://dotwa.org.au/v2/wp‐content/uploads/2018/03/DOTWA‐DCD‐Info‐Sheet.pdf	Distribution of research findings and links to resources via the same channels used to distribute this survey (e.g., via specific social media groups such as Practical Parenting, Parent Talk Australia and Smart Parenting)

All stakeholders in this study play a unique and important role in the identification of children with DCD. Improved familiarity and knowledge of the disorder is needed for access to appropriate services and improved long‐term outcomes for this condition. First, parents and teachers need to identify delays in motor skills acquisition so that they might seek professional assistance. Allied health professionals currently have the highest levels of familiarity and knowledge and should therefore play a part in educating others, in addition to their role in identifying the disorder. Ultimately, medical professionals must make a diagnosis so that children and families might receive appropriate support and services.

## 6 STRENGTHS AND LIMITATIONS

To our knowledge, this is the first study of the awareness and knowledge of DCD in the Australian population. Although results may not be directly generalizable to other countries, the findings are similar to those of Wilson et al. (2012) which included stakeholders from the United States, the United Kingdom and Canada. The overall sample size in this study was small, particularly in regard to medical professionals, which may reflect the level of interest in completing a survey of DCD. Although the majority of respondents were from Western Australia, there was representation from each state in Australia and in most stakeholder groups.

There was a disproportionate number of occupational therapists in the allied health group; however, this is consistent with recent findings that most Australian families who accessed therapy for their child's movement difficulties had seen an occupational therapist (79.5%), compared with 45.5% seeing a physiotherapist and 19.6% seeing a specialized exercise physiologist (Licari et al., 2020).

Most participants chose to comment in the open‐ended question; however, respondents choosing to answer the open‐ended questions may be systematically different from the respondents overall because of the nature of self‐selection.

## 7 RECOMMENDATIONS FOR CLINICAL PRACTICE AND FUTURE RESEARCH

Improved knowledge translation should be a priority for clinicians and researchers in the field of DCD. A key knowledge translation principle for clinicians is to monitor knowledge use (Graham et al., 2006) which can be achieved with clinical audits to monitor diagnosis, assessment and intervention of children with DCD. Future research in this field should explore barriers to knowledge use across all stakeholders.

## ETHICS STATEMENT

This research received ethical approval through Edith Cowan University, Western Australia (approval number: 2019‐00106‐HUNT). We were not funded for this research and the findings of this research have not been presented at this time.

## Supporting information


**Data S1.** Supporting Information
