# Supplementary material for: Awareness and knowledge of developmental coordination disorder: A survey of caregivers, teachers, allied health professionals and medical professionals in Australia
Source: Child Care Health Dev. 2020 Nov 16;47(2):174–83. doi: 10.1111/cch.12824 (PMC7894302; doi:10.1111/cch.12824)
Supplement: Supplementary file 1 — Data S1. Supporting Information [file CCH-47-174-s001.docx]

**Appendix A**

**Australian Knowledge and Perceptions of DCD**

Participant Information and Consent Form
*Developmental Coordination Disorder: Knowledge and perceptions of key stakeholders in Australia*

**Contact details of researchers:**

Jacqui Hunt

Lecturer

School of Medical and Health Sciences

Edith Cowan University

Ph: +61 8 6304 3582

Email: j.hunt@ecu.edu.au

Associate Professor Annette Raynor

School of Medical and Health Sciences

Edith Cowan University

Ph: +61 8 6304 2771

Email: a.raynor@ecu.edu.au

Associate Professor Erin Godecke

School of Medical and Health Sciences

Edith Cowan University

Ph: +61 8 6304 5901

Email: e.godecke@ecu.edu.au

Associate Professor Jill Zwicker

Department of Occupational Science and Occupational Therapy

The University of British Columbia

Ph: 0011 1 604 875 2345

Email: jill.zwicker@ubc.ca

**Introduction**This information sheet provides a description of the study “*Developmental Coordination Disorder: Knowledge and perceptions of key stakeholders in Australia*." Please take the time to read this information carefully for your understanding, and please forward the details of this study to anyone you think may be eligible to participate. Please note that participation in this study is entirely voluntary and in selecting “I agree to participate in this study,” you are indicating your consent.

**Purpose of the study**To explore the knowledge and perceptions about Developmental Coordination Disorder (DCD) among caregivers, teachers, allied health and medical professionals to identify possible barriers to diagnosis. This research will add to the knowledge-base associated with DCD, and findings from this study will support further research.
Who can participate in this study
Parents/caregivers: If you are a primary caregiver of a child/children (with or without DCD) aged 0 to 19 years and you reside in Australia.
OR
Teachers: If you have current and valid registration with the teaching board in your state to teach in Australian schools and if you teach/have taught children (with or without DCD) between 0 and 19 years.
OR
Allied health professionals (AHP): If you have current and valid registration with the Australian Health Practitioner Regulation Agency (AHPRA) or equivalent professional recognition to work as an AHP in Australia, and your practice comprises of/or has previously comprised of at least 15% children (with or without DCD) aged between 0 and 19 years.
OR
Medical professionals: If you have current and valid registration with the Medical Board of Australia to work as a medical professional in Australia, and your practice comprises of/or has previously comprised of at least 15% children (with or without DCD) aged between 0 and 19 years.
  
Note: The age range of children is based on the World Health Organisation’s classification of individuals below the age of adulthood.
  
What participation involves and how long it will take
Once you have selected “I agree to participate in this study,” you will commence the online questionnaire which will take approximately 5-10 minutes to complete.
  
**Conduct**This research study is conducted as part of a larger research project by Jacqui Hunt, towards the requirement of completion of a Doctorate of Philosophy (PhD).
  
**Privacy and confidentiality**All information relating to this research project will remain confidential. Electronic data will be stored on a password protected computer for the duration of this project, and any hard-copy materials will be kept in a locked office at Edith Cowan University.

**What happens with the results**Results of this study may be published in a journal, communicated with relevant stakeholders, and/or disseminated at conference presentations. Results will be presented in a manner that ensures confidentiality of participants.

**What happens upon completion of the study**The survey will be accessible for six weeks. After this time, data will be analysed to determine the knowledge and perceptions of DCD among the four participant groups. If you are interested, a summary of the results and a copy of the published journal article may be sent to you upon completion of the study. Please email AUTHOR NAME IDENTIFICATION REMOVED at E-MAIL IDENTIFICATION REMOVED University to request this information.
Storage of data will adhere to the IDENTIFICATION REMOVED University regulations whereby data are stored securely for a minimum of seven years after completion of the project. All electronic data will be transferred to an external hard drive in a password protected file and stored by a supervisor of this research project. After the seven-year period, all electronic data will be erased, and all hard-copy materials will be shredded.
  
**Potential benefits and risks of participation**There are no risks associated with participation in this study. Some participants may experience minor inconvenience when completing the online survey. Benefits of participating in this study involve gaining greater awareness and knowledge about DCD.
  
**Extent to which participation is voluntary**Participation in this study is entirely voluntary and participants may choose to withdraw from the study at any point in the research process with no implications. An option to exit the survey is included at the bottom of every section in the questionnaire, allowing participants to exit at any time if they choose to do so.
  
Once data have been published, participants’ contribution to the study cannot be removed from the publication. Requests for withdrawal from the study should be made prior to publishing.
Who to contact regarding questions and/or concerns
Contact details of the researchers are provided at the top of this information sheet. Please contact Jacqui Hunt if you have any questions or concerns related to this study. Alternatively, you may wish to contact the principal supervisor of this research project: Associate Professor Annette Raynor.

**Ethics Approval**Approval for this study has been obtained from the Human Research Ethics Committee of Edith Cowan University (Approval number: 2019-00106-HUNT). If you have any queries or concerns regarding the way in which this research is conducted, please contact:
  
Research Ethics Officer

Edith Cowan University

Joondalup Campus

270 Joondalup Drive

JOONDALUP WA 6027

Ph: 6304 2170

Email: research.ethics@ecu.edu.au

Thank you for taking the time to read this information and consider participation in this study. If you wish to participate, please read the declaration on the following page, select “I agree to participate in this study," and you will commence the questionnaire. If you do not wish to participate in this study, select "I decline to participate in this study."

**Participant declaration**

- I confirm that I have read and understood the information provided to me regarding the aims, process, and risks of this research project.
- I am willing to participate in this study by completing the online questionnaire, as described.
- I understand that participation in this study is entirely voluntary and that I have the right to withdraw from this study at any point in the process up until data are published.
- I understand that all data collected and information provided will be strictly confidential.
- I understand that this research may be published in a journal, communicated with relevant stakeholders, and/or disseminated at conference presentations.
- I agree to the sharing of the research findings, knowing that the information I supply remains confidential.
- I agree to participate in this study
- I decline to participate in this study

*The following survey has been adapted from Wilson, Neil, Kamps, and Babcock (2013) with the permission of the author.*

In which state/territory do you reside?

- Australian Capital Territory
- New South Wales
- Northern Territory
- Queensland
- South Australia
- Tasmania
- Victoria
- Western Australia
- I do not live in Australia

Have you previously completed a survey regarding your knowledge of Developmental Coordination Disorder?

- Yes
- No

Please indicate your gender:

- Male
- Female
- Other

Are you a(n) *(select all that apply):*

- Primary caregiver
- Teacher
- Allied health professional
- Medical professional

**The following questions relate to your role as a caregiver:**

What age is/are your own child/children? *(you may select more than 1 box):*

- 0-2 years
- 3-5 years
- 6-12 years
- 13-19 years
- 20+ years

**The following questions relate to your role as an allied health professional:**

As an allied health professional, do you have current and valid registration with the Australian Health Practitioner Regulation Agency (AHPRA) or equivalent professional recognition to work as an allied health professional in Australia?

- Yes
- No *(If no is selected, no further questions regarding your allied health profession will be asked)*

As an allied health professional, does your caseload consist of (or has your caseload ever consisted of) at least 15% children (0 to 19 years)?

- Yes
- No *(If no is selected, no further questions regarding your allied health profession will be asked)*

What is your allied health profession?

- Occupational Therapist
- Physiotherapist
- Speech Pathologist
- Psychologist
- Social worker
- Other

Please type your allied health profession in the space below

________________________________________________________________

Where did you receive the qualification you require to practice as an allied health professional?

- Australian Capital Territory
- New South Wales
- Northern Territory
- Queensland
- South Australia
- Tasmania
- Victoria
- Western Australia
- Outside Australia

In which country did you receive the qualification you require to practice as an allied health professional?

________________________________________________________________

How many years have you worked as an allied health professional? *(Please type your answer using numerical symbols e.g., "5" not "five"):*

________________________________________________________________

In which of these locations have you worked as an allied health professional with children between 0 and 19 years of age? *(select all that apply):*

- Australian Capital Territory
- New South Wales
- Northern Territory
- Queensland
- South Australia
- Tasmania
- Victoria
- Western Australia
- Outside of Australia

Please list any other countries (outside of Australia) in which you have worked as an allied health professional with children between 0 and 19 years of age*:*

________________________________________________________________

**The following questions relate to your role as a medical professional:**

As a medical professional, do you have current and valid registration with the Medical Board of Australia to work as a medical professional in Australia?

- Yes
- No (If no is selected, no further questions regarding your medical profession will be asked)

As a medical professional, does your caseload consist of (or has your caseload ever consisted of) at least 15% children (0 to 19 years)?

- Yes
- No *(If no is selected, no further questions regarding your medical profession will be asked)*

What is your profession as a medical practitioner?

General Practitioner

Paediatrician

Other

Please type your medical profession in the space below

_______________________________________________________________

Where did you receive the qualification you require to register as a medical professional?

- Australian Capital Territory
- New South Wales
- Northern Territory
- Queensland
- South Australia
- Tasmania
- Victoria
- Western Australia
- Outside of Australia

How many years have you worked as a medical professional? *(Please type your answer using numerical symbols e.g., "5" not "five"):*

________________________________________________________________

Please type the country in which you received the qualification you require to register as a medical professional?

________________________________________________________________

In which of these locations have you worked as a medical professional with children between 0 and 19 years of age? *(select all that apply):*

- Australian Capital Territory
- New South Wales
- Northern Territory
- Queensland
- South Australia
- Tasmania
- Victoria
- Western Australia
- Outside of Australia

Please list any other countries (outside of Australia) in which you have worked as a medical professional with children between 0 and 19 years of age*?:*

________________________________________________________________

________________________________________________________________

**The following questions relate specifically to your time working with children aged 0-19 years of age**

In a standard week, approximately how many children (between 0 and 19 years) would be seen in your practice? *(Please type your answer using numerical symbols E.g. "5" not "five")*

________________________________________________________________

In a standard week, approximately how many children (between 0 and 19 years) would be seen ***on your caseload?*** *(Please type your answer using numerical symbols e.g., "5" not "five")*

________________________________________________________________

In a standard week, how many children would be seen ***in your own caseload*** that would be described by the following conditions? *(Please type your answer using numerical symbols e.g., "5" not "five")*

- Has evidence of difficulty learning (learning disability) ____________________
- Demonstrates oppositional behaviour _________________________
- Is self-conscious or stressed about his or her physical skills _________________________
- Has trouble ‘keeping up’ with other kids in physical games or sports _________________
- Cannot complete tasks such as writing, drawing, or handling small objects, in a way that’s adequate for their age ___________________
- Appears to be clumsy and accident prone when compared to their peers _______________
- Has difficulty moving their mouth in such a way that their speech is easy to understand, yet passes standard speech tests ____________________________

As an allied health professional, have you ever identified probable DCD using standardised assessments?

- Yes
- No

As a medical professional, have you diagnosed a child with DCD?

- Yes
- No

As a teacher, do you have current and valid registration with your relevant state or territory authority?

- Yes
- No (If no is selected, no further questions regarding your teaching profession will be asked)

**The following questions relate to your role as a teacher:**

In your role as a teacher, what is the age range of your current/past students? *(select all that apply)*:

- 0-2 years
- 3-5 years
- 6-12 years
- 13-19 years
- 20+ years

Where did you receive the qualifications you required to register as a teacher?

- Australian Capital Territory
- New South Wales
- Northern Territory
- Queensland
- South Australia
- Tasmania
- Victoria
- Western Australia
- Outside Australia

Please type the country in which you received  the qualifications you required to register as a teacher:

________________________________________________________________

How many years have you worked as a teacher? *(Please type your answer using numerical symbols e.g., "5" not "five")*

________________________________________________________________

In which of these locations have you taught children between 0 and 19 years? *(select all that apply)*:

- Australian Capital Territory
- New South Wales
- Northern Territory
- Queensland
- South Australia
- Tasmania
- Victoria
- Western Australia
- Outside of Australia

Please list any other countries (outside of Australia) in which you have taught children between 0 and 19 years of age:

___________________

Approximately how many students do you have in your school? *(Please type your answer using numerical symbols e.g., "5" not "five").*

__________________

Approximately how many children would be taught in your class?  *(Please type your answer using numerical symbols e.g., "5" not "five").*

__________________

Approximately how many children would be taught **in your class** that would be described by the following conditions?*(Please type your answers using numerical symbols)*

- Has evidence of difficulty learning (learning disability) __________________
- Demonstrates oppositional behaviour ________________
- Is self-conscious or stressed about his or her physical skills ________________
- Has trouble ‘keeping up’ with other kids in physical games or sports _______________
- Cannot complete tasks such as writing, drawing, or handling small objects, in a way that’s adequate for their age ________________________________________________
- Appears to be clumsy and accident prone when compared to their peers _______________
- Has difficulty moving their mouth in such a way that their speech is easy to understand, yet passes standard speech tests ____________________

How familiar are you with the following conditions?

|  | I have not heard of this condition at all | Very unfamiliar | Somewhat unfamiliar | Somewhat familiar | | Very familiar |
| --- | --- | --- | --- | --- | --- | --- |
| Obsessive Compulsive Disorder (OCD) |  |  |  |  |  | |
| Asperger’s Syndrome |  |  |  |  |  | |
| Global Developmental Delay |  |  |  |  |  | |
| Conduct Disorder |  |  |  |  |  | |
| Clumsy Child Syndrome |  |  |  |  |  | |
| Attention Deficit Hyperactivity Disorder (ADHD) |  |  |  |  |  | |
| Spina Bifida |  |  |  |  |  | |
| Dyspraxia |  |  |  |  |  | |
| Mental Retardation |  |  |  |  |  | |
| Dyslexia |  |  |  |  |  | |
| Developmental Coordination Disorder (DCD) |  |  |  |  |  | |
| Oppositional Defiance Disorder (ODD) |  |  |  |  |  | |
| Autism |  |  |  |  |  | |
| Motor Learning Disability |  |  |  |  |  | |
| Chromosomal Disorders |  |  |  |  |  | |
| Autism Spectrum Disorder (ASD) |  |  |  |  |  | |
| Learning Disability |  |  |  |  |  | |
| Intellectual Disability |  |  |  |  |  | |

To your knowledge, which of the following do you think are part of the condition of Developmental Coordination Disorder (DCD) / Clumsy Child Syndrome / Dyspraxia / Motor Learning Disability?

|  | Common feature of the condition of DCD | May be a feature of the condition of DCD | **Not** part of the condition of DCD | Unsure |
| --- | --- | --- | --- | --- |
| Motor learning difficulties. |  |  |  |  |
| Difficulty printing and/or writing. |  |  |  |  |
| Gross motor and/or fine motor skills delay. |  |  |  |  |
| Low self-esteem. |  |  |  |  |
| Poor physical fitness. |  |  |  |  |
| Sensory processing challenges. |  |  |  |  |
| Anxiety |  |  |  |  |
| Difficulty making friends. |  |  |  |  |
| Poor social skills. |  |  |  |  |
| Depression. |  |  |  |  |
| Poor academic performance. |  |  |  |  |
| Average (or above average) cognitive ability. |  |  |  |  |
| Below average cognitive ability. |  |  |  |  |
| Higher than average risk for suicide. |  |  |  |  |
| Obesity. |  |  |  |  |

As a caregiver, do you agree or disagree with the following statements relating to children and Developmental Coordination Disorder (DCD)?

|  | Agree | Disagree | Unsure |
| --- | --- | --- | --- |
| I believe education and health care should work together to help identify children with DCD. |  |  |  |
| Children with DCD should be supported by trained professionals in the school system. |  |  |  |
| I feel there should be more education for parents about the signs of DCD. |  |  |  |
| I feel confident that if my child had DCD, my child’s physician would be able to provide an accurate diagnosis in a timely manner. |  |  |  |
| I feel confident that if my child had DCD, I would be able to tell. |  |  |  |
| I believe there are adequate resources in place to support children with DCD. |  |  |  |
| I would hesitate to have my child diagnosed with DCD because I wouldn’t want them to be ‘labelled.’ |  |  |  |

What are the major factors that influenced your answers to the above questions?

________________________________________________________________

________________________________________________________________

________________________________________________________________

As an allied health or medical professional, do you agree or disagree with the following statements relating to children and Developmental Coordination Disorder (DCD)?

|  | Agree | Disagree | Unsure |
| --- | --- | --- | --- |
| Further research is needed on DCD |  |  |  |
| I feel I need more education/ information regarding the condition of DCD. |  |  |  |
| I believe there are significant benefits from an accurate diagnosis of DCD being given early. |  |  |  |
| Learning that the estimated incidence of DCD is between 5% and 6% in children would surprise me. |  |  |  |
| The DSM-5 contains enough information about DCD for an accurate diagnosis to be made. |  |  |  |
| DCD would be relatively easy to identify. |  |  |  |

What are the major factors that influenced your answers to the above questions?

________________________________________________________________

________________________________________________________________

________________________________________________________________

As a teacher, do you agree or disagree with the following statements relating to children and Developmental Coordination Disorder (DCD)?

|  | Agree | Disagree | Unsure |
| --- | --- | --- | --- |
| Accurate diagnoses and classifications are critical for educators to know how to help children with DCD. |  |  |  |
| I believe educators should play a role in identifying early warning signs that can help to diagnose DCD. |  |  |  |
| Trying to manage children with DCD in the classroom is overwhelming. |  |  |  |
| There are too many conditions for educators to keep up with. |  |  |  |
| Currently, the education system would not be able to adequately support children with DCD due the lack of knowledge and perceptions about the condition. |  |  |  |
| I believe there are children in the school system labelled as lazy or defiant that in fact have gross and/or fine motor skills impairments. |  |  |  |
| There are adequate support professionals for children with DCD in the school system. |  |  |  |

What are the major factors that influenced your answers to the above questions?

________________________________________________________________

________________________________________________________________

________________________________________________________________

As a caregiver, if you have any concerns for your child regarding Developmental Coordination Disorder, please visit your family doctor.
